# Imported malaria in a non-endemic country: sixteen years of cases in a hospital in the South of Madrid, Spain

**DOI:** 10.1007/s10096-024-04938-0

**Published:** 2024-09-17

**Authors:** Víctor Antón-Berenguer, Irene Cabrera-Rodrigo, Beatriz Valle-Borrego, Jorge Ligero-López, Francisco J. Merino-Fernández, Sara Gómez-de-Frutos, José Miguel Rubio

**Affiliations:** 1 Severo Ochoa University Hospital, Avenida de Orellana s/n Leganés, Madrid, 28911 Spain; 2 Faculty of Medicine, Alfonso X el Sabio University, Madrid, Spain; 3 Department of Microbiology, Faculty of Medicine, Paediatrics, Radiology and Public Health, Zaragoza University, Zaragoza, Spain; 4 Malaria & Emerging Parasitic Diseases Laboratory, Parasitology Department, National Centre of Microbiology. Instituto de Salud Carlos III, Cra. Majadahonda Pozuelo Km.2, Majadahonda, Madrid, 28220 Spain

**Keywords:** Malaria, VFR, Migrants, Coinfections, Semi-immunity, Submicroscopic malaria

## Abstract

**Purpose:**

Malaria keeps on being a serious global health threat, especially in many tropical countries, where it is endemic. Also in non-endemic countries, like Spain, malaria is an issue that requires attention due to the presence of imported cases.

**Methods:**

This is a retrospective study, including all patients diagnosed with malaria at Severo Ochoa University Hospital from 2006 to 2022, being classified according to: (I) their type of stay in an endemic area as visiting friends and relatives (VFR), migrants of recent arrival (MRA), or tourism and business (T&B), and (II) the mode of presentation as microscopic (MM) or submicroscopic (SMM) malaria.

**Results:**

In this study, 132 patients (23.7% of all suspected) were diagnosed with malaria. The PCR was the most sensitive technique (99.2%), followed by antigen detection (78.8%) and microscopy (75%), with *Plasmodium falciparum* being the predominant species (94.7%). VFR was the largest group infected with malaria (69.7%), mostly symptomatic (98.2%) and presenting MM (90.2%). Instead, MRA patients (25%) presented milder (47.4%) or no symptoms (31.6%) and higher cases of SMM (42.4%). Coinfection with another imported pathogen was present in 19 patients (14.4%), being MRA more frequently coinfected (30.3%)

**Conclusion:**

This study shows the need for establishing systems for VFRs to attend pre-travel consultations to reduce malaria imported risk. In the case of MRA, screening for imported diseases should be conducted upon their arrival. Finally, we highlight two cases of co-infection with imported viruses, showing that presence of symptoms resembling malaria from another imported pathogen does not exclude malaria.

## Background

Even with malaria´s morbidity and mortality diminution in the last decades, it keeps on being a serious global threat. According to the last WHO malaria report, 247 million cases occurred globally in 2021, continuing an upward trend that started in 2016 which seems to remain stable despite the impact of COVID-19 [1]. Oppositely, in Europe, due to the imported nature of malaria cases and the decrease in international travel during 2020, an important diminution in malaria cases was reported during that year, occurring a slight case rise in 2021, and being Spain one of the countries that notified a higher number of malaria cases [2].

All malaria cases notified in our country, since malaria eradication in 1964, have been imported, with some exceptions: two cases of airport malaria [3, 4]; five neonatal malaria cases, and some sporadic cases related to the sanitary environment (transfusions, organ transplantation or parenteral transmission) [5–7]. Finally, two cases of vectorial paludism, being caused by *P. vivax* were notified in 2010 and 2014 [8, 9].

Even though malaria diagnostic techniques have changed in the last decades, thick blood microscopic examination keeps on being the golden standard [10] but antigenic system detection has been extended (Rapid Diagnostic Tests), especially in endemic countries. Molecular methods such as PCR or qPCR have shown to be more sensitive [11], being able to detect submicroscopic malaria (SMM) infections, presenting with a low density of *Plasmodium* hematic forms that make it not detectable by conventional microscopy. These SMM cases, despite being minoritarian, suppose a percentage ranging between 5% and 25% of all cases in Spain [12–14].

In this study, we reviewed the malaria cases diagnosed from January 2006 to December 2022 in the Severo Ochoa University Hospital (SOUH), a secondary hospital in the South of Madrid (Spain), analysing the sociodemographic profile of malaria patients, the context of its infection, illness presentation and accuracy of the diagnostic methods and treatments employed, comparing some key issues of the illness among three different population groups (VFR, MRA and T&B), as well as differences between SMM and MM patients.

## Methods

### Population and study period

All patients who attended SOUH’s emergency department with suspected malaria from January 2006 to December 2022 were included in this retrospective study. SOUH is the reference hospital for the municipality of Leganés (Madrid, Spain) with a population of 186.660 inhabitants, in January 2022, from whom 15.34% were migrants. Beyond the migrant population, 58.2% was originally from South America, 16.4% from Africa and 4.7% from Asia.

Patients were classified by the type of stay in an endemic area or by the mode of presentation of malaria. In the first case, they are classified into three groups; (i) MRA: migrants of recent arrival who leave the endemic area for the first time; (ii) VFR: migrants living in Spain who travel to their original countries to visit their relatives and friends; and, (iii) T&B: non-endemic area born patients who travel to endemic areas for tourism or business reasons. Depending on the mode of presentation the patients were classified into two groups: microscopic malaria cases (MM), which were detected either by microscopy or antigenic detection and submicroscopic malaria cases (SMM), which were only detected by a Nested Multiplex PCR (NM-PCR).

### Epidemiological survey

Analysed data included: admission date, age, sex, birth and residence country, travel to an endemic area, country of the travel, length of stay, motive of the travel, performance or not of chemoprophylaxis, haemoglobin, haematocrit, platelets, leukocyte, bilirubin levels, diagnostic method, *Plasmodium* species, parasitemias, coinfection with another imported disease, clinical presentation, treatment and hospitalization.

Data were obtained from the clinical records of the patients as well as the Microbiology Department database for all those patients who met the malaria case definition, which was established based on the patient’s clinical signs and exposure factors accompanied by a microbiological confirmation.

### Diagnostic procedures

The microbiological diagnosis was performed through (i) microscopy (Giemsa-stained thick/thin smear); (ii) immunochromatography-based antigen detection rapid tests (ICT), using BinaxNOW™ test (Abbot) until 2008 and Biosynex^®^ MALARIA *P.f*/Pan (Biosynex) after 2008; and (iii) molecular diagnosis by Malaria PCR (NM-PCR) carried out in the Spanish malaria reference laboratory of the Microbiology National Centre at the Instituto de Salud Carlos III [15].

### Statistical analysis

Statistical analysis was performed employing chi-square or t-student, using the most accurate depending of the parameter, showing p values and taking as statistically significant each comparison with p *< 0.05*.

When analysing the different parameters studied, patients whose data were not available were not considered for the calculi. In addition, patients presenting more than one different coinfection, symptoms or analytical alterations were considered more than once.

### Ethics

This study has the approval of the Clinical Research Ethics Committee of Severo Ochoa University Hospital C.E.I.m A-1493-2023.

## Results

**Fig. 1:**
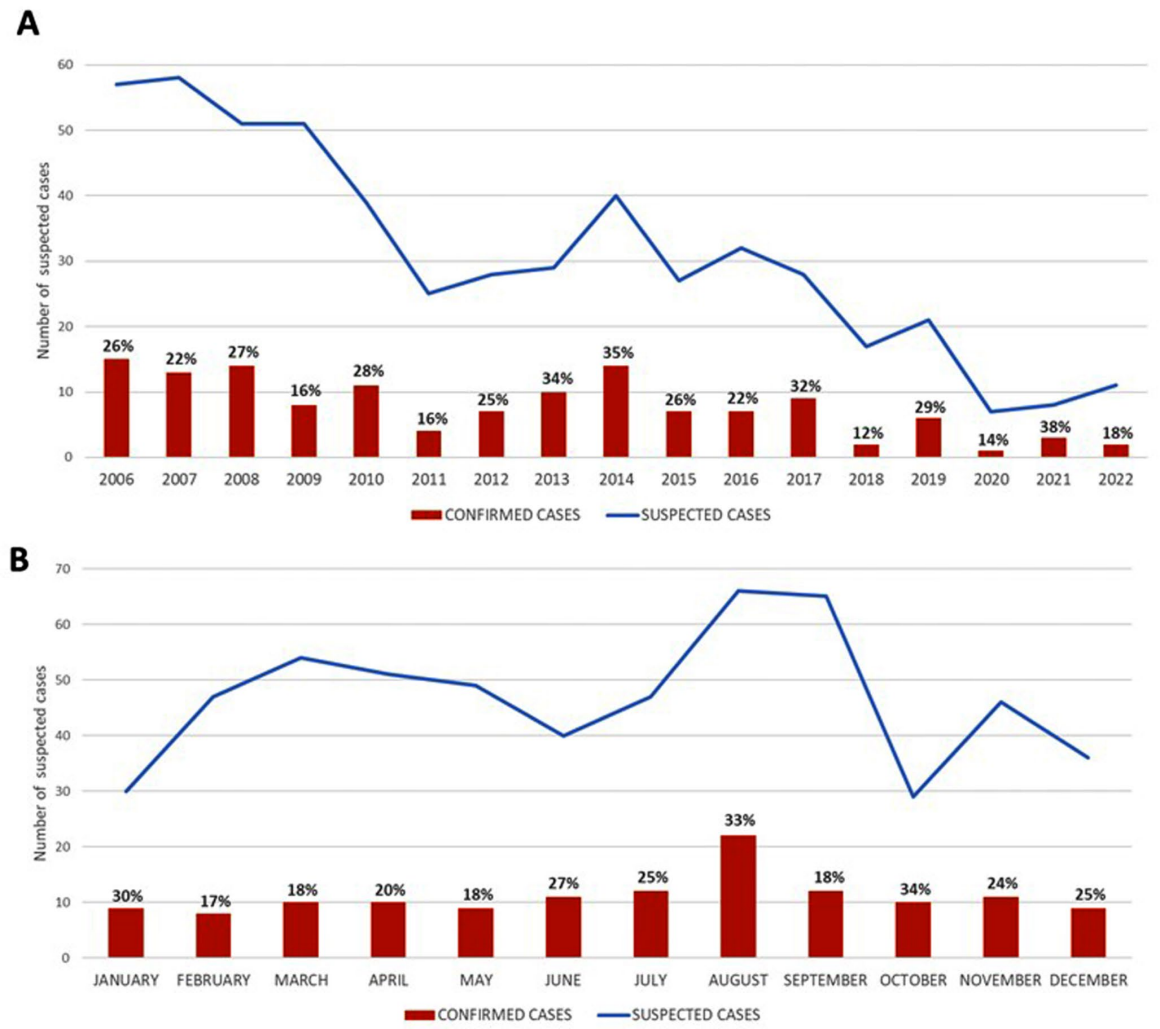
Inter-annual (**A**) and intra-annual (**B**) distribution of malaria suspected and confirmed cases. At the top of each bar, positivity percentages are found

In the period studied, 557 patients attended SOUH´s emergency department with suspected malaria, 283 (50.8%) men, and 274 (49.2%) women, of which 132 (23.7%) were malaria positive, 61 (46.2%) male and 71 (53.8%) female. The presentation of cases does not have an accumulation for years but does preferably occur in the summer months, especially in August (Fig. 1). Depending on the diagnostic method used, 99 (75%), 104 (78.8%) and 131 (99.2%) positive cases were characterized by microscopy, ICT and NM-PCR respectively.

*Plasmodium falciparum* was the predominant species characterised, being the causal agent of 125 (94.7%) cases, followed by *P. ovale* with 5 cases (3.8%) and *P. malariae* with 2 cases (1.5%). No cases of malaria due to *P. vivax* or *P. knowlesi* were detected.

Twenty-five out of 132 cases (18.9%) were detected only by NM-PCR, so they were considered SMM. One case was detected only by microscopy neither by NM-PCR nor by ICT. The 99 positive cases detected by microscopy were considered microscopic malaria (MM), parasitemia was determined in 93 of them; 89 *P. falciparum*, 3 *P. ovale* and 1 *P. malariae*. *Plasmodium falciparum* showed a mean of 49 092 trophozoites/µl (range 100–430 000 trophozoites/µl), while for non-*falciparum* species the parasitemia mean was 16 150 trophozoites/µl (range: 2 600 − 36 000 trophozoites/µl).

By type of stay in an endemic area, VFR patients, who were living in Spain for a mean of 10 years (ranging between 1.5 months and 37 years), resulted to be the largest group, with 92 cases (69.7%), followed by MRA, with 33 patients (25%) and finally, T&B, with 7 patients (5.3%). In those patients who got malaria during a trip (VFR and T&B) the time they stayed in an endemic area ranged from 4 to 421 days, with a mean of 57 days (Table 1).

**Table 1:** Epidemiological, clinical and diagnostic parameters of malaria patients, divided by travel motive

	Total	VFR	MRA	T&B	ρ
N	132	92(69.7%)	33(25%)	7(5.3%)	*-*
Age (mean)	36	35	25	48	***0.005***
*Sex*					
Male	61(46.2%)	43(46.7%)	12(36.4%)	6(85.7%)	***0.05***
Female	71(53.8%)	49(53.26%)	21(63.6%)	1(14.29%)	***0.04***
*Infection country*					
Equatorial Guinea	62 (47.2%)	36(39.1%)	23(69.7%)	3(50%)	***0.01***
Nigeria	48(36.6%)	40(43.5%)	8(24.2%)	0(0%)	***0.04***
Cameroon	12(9.2%)	10(10.8%)	2(6.1%)	0(0%)	*0.5*
Angola	2(1.5%)	1(1.1%)	0(0%)	1(16.7%)	*-*
Ivory Coast	2(1.5%)	2(2.17%)	0(0%)	0(0%)	*-*
Mali	2(1.5%)	2(2.17%)	0(0%)	0(0%)	*-*
Liberia	1(0.76%)	1(1.1%)	0(0%)	0(0%)	*-*
India	1(0.76%)	0(0%)	0(0%)	1(16.7%)	*-*
Zambia	1(0.76%)	0(0%)	0(0%)	1(16.7%)	*-*
Unknown	**1(0.8%)**	**0(0%)**	**0(0%)**	**1(14.3%)**	***-***
*Plasmodium species*					
*P. falciparum*	125(94.7%)	88(95.6%)	31(93.9%)	6(85.7%)	*0.6*
*P. ovale*	5(3.8%)	3(3.2%)	1(3.1%)	1(14.3%)	*0.3*
*P. malariae*	2(1.5%)	1(1.1%)	1(3%)	0(0%)	*-*
*P. vivax*	0(0%)	0(0%)	0(0%)	0(0%)	*-*
*Coinfections*					
Total	19(14.4%)	8(8.7%)	10(30.3%)	1(14.3%)	***0.01***
Filaria	8(6.1%)	3(3.2%)	4(12.1%)	1(14.3%)	***0.1***
Geohelminths	10(7.6%)	2(2.2%)	8(24.2%)	0(0%)	***< 0.001***
Flukes	4(3%)	1(1.1%)	3(9.1%)	0(0%)	***0.03***
Protozoa	3(2.3%)	1(1.1%)	2(6.1)	0(0%)	*0.1*
Virus	2(1.5%)	2(2.2%)	0(0%)	0(0%)	*-*
Poliparasitized (+ 2)	7(5.3%)	2(2.2%)	5(15.2%)	0(0%)	***0.01***
Not coinfected	**113(85.6%)**	**84(91.3%)**	**23(69.7%)**	**6(85.7%)**	***-***
*Symptoms*					
Symptomatic	71(91%)	5 (98.2%)	13(68.4%)	4(100%)	***0.004***
Fever	49(69%)	37(67.3%)	8(42.1%)	4(100%)	*0.04*
Diarrhoea	12(15.4%)	9(16.4%)	2(10.5%)	1(25%)	*0.7*
Abdominal pain	16(22.5%)	13(23.6%)	2(10.5%)	1(25%)	*0.4*
Secondary symptoms^1^	55(77.5%)	43(78.2%)	9(47.4%)	3(75%)	*0.03*
Asymptomatic	7(9%)	1(1.8%)	6(31.6%)	0(0%)	***0.004***
Unknown	**54(40.9%)**	**37(40.2%)**	**14(42.4%)**	**3(42.9%)**	***-***
*Type of malaria*					
MM	107(81.1%)	83(90.2%)	19(57.6%)	5(71.4%)	***< 0.001***
< 2.500 trophozoites/µl	12(12.9%)	10(13.3%)	2(15.4%)	0(0%)	*0.8*
2.500–150.000 trophozoites/µl	75(80.6%)	60(80%)	10(76.9%)	5(71.4%)	*0.5*
> 150.000 trophozoites/µl	6(6.5%)	5(6.7%)	1(7.7%)	0(0%)	*0.8*
Not determined	**6(6.1%)**	**3(3.8%)**	**3(18.8%)**	**0(0%)**	***-***
SMM	25(18.9%)	9(9.8%)	14(42.4%)	2(28.6%)	***< 0.001***
*Diagnosis delay*					
< 30 days	93(76.2%)	73(81.8%)	18(60%)	3(75%)	*0.06*
30 days-3 months	18(14.8%)	10(12.5%)	7(24.1%)	0(0%)	*0.1*
> 3 months	11(9%)	5(5.7%)	5(17.2%)	1(25%)	*0.1*
Unknown	**10(7.6%)**	**4(5.6%)**	**3(9.4%)**	**3(42.9%)**	***-***
*Chemoprophylaxis*					
Correct	3(2.3%)	2(2.2%)	0(0%)	1(14.3%)	*0.1*
Incorrect	8(6.1%)	7(7.6%)	0(0%)	1(14.3%)	*0.4*
None	121(91.7%)	83(90.2)	33(100%)	5(71.4%)	*0.1*
*Analytic alterations*					
Anaemia (Hb < 11.6 g/dL for women, Hb < 13.2 g/dLfor men)	47(20.3%)	34(37%)	13(39.4%)	0(0%)	*0.8*
Leucocytosis (> 4.8*10^3/µL)	74(56.1%)	53(57.6%)	15(45.4%)	6(85.7%)	*0.1*
Thrombocytopenia (< 130*10^3/µL)	0(0%)	0(0%)	0(0%)	0(0%)	*-*
GPT (> 45 U/L)	13(9.8%)	10(10.1%)	1(3%)	2(28.6%)	*0.1*
GOT (> 34 U/L)	20(15.2%)	14(15.2)	3(9.1%)	3(42.9%)	*0.07*
Bilirubin (< 1.2 mg/dL)	18(13.6%)	15(16.3%)	2(6.1%)	1(14.3%)	*0.3*

*VFR: Visiting Relatives and Friends*,* MRA: Migrants of recent arrival*,* T&B: Tourism and Business*,* MM: Microscopical Malaria*,* SMM: Submicroscopic Malaria*,* Hb: Haemoglobin*,* GPT: Glutamine-pyruvate transaminase*,* GOT: Glutamine-oxaloacetate transaminase*

^*1*^*Secondary symptoms include vomiting*,* headache*,* cough*,* shivering*,* asthenia and general discomfort*

By species, 30 cases out of 125 (24%) of *P. falciparum*, two out of five (40%) of *P. ovale* cases and one out of two (50%) of *P. malariae* cases were considered SMM. Fifteen out of the 30 SMM *P. falciparum* cases occurred in migrants of recent arrival (MRA) as the SMM case of *P. malariae* and one out of two *P. ovale* SMM cases (Table 2).

**Table 2:** Epidemiological, clinical and diagnostic parameters of malaria patients, divided by malaria type presented

	TOTAL	SMM	MM	ρ
N	132	25(18.9%)	107(81.1%)	
Age (mean)	36	30	34	***0.2***
***Sex***				
Male	61(46.2%)	11(44%)	50(46.7%)	*0.8*
Female	71(53.8%)	14(56%)	57(53.3%)	*0.7*
***Travel motive***				
VFR	92(69.7%)	9(36%)	83(77.6%)	***< 0.001***
MRA	33(25%)	14(56%)	19(17.7%)	***< 0.001***
TOURISTS	7(5.3%)	2(8%)	5(4.7%)	*0.5*
***Infection country***				
Equatorial Guinea	62(47.2%)	14(56%)	48(44.9%)	*0.3*
Nigeria	48(36.6%)	7(28%)	41(38.2%)	*0.3*
Cameroon	12(9.2%)	1(4%)	11(10.3%)	*0.3*
Angola	2(1.5%)	0(0%)	2(1.9%)	*-*
Ivory Coast	2(1.5%)	0(0%)	2(1.9%)	*-*
Mali	2(1.5%)	0(0%)	2(1.9%)	*-*
Liberia	1(0.76%)	1(4%)	0(0%)	*-*
India	1(0.76%)	1(4%)	0(0%)	*-*
Zambia	1(0.76%)	1(4%)	0(0%)	*-*
**Unknown**	**1(0.8%)**	**0(0%)**	**1(0.9%)**	*-*
***Plasmodium species***				
*P. falciparum*	125(94.7%)	22(88%)	103(92.3%)	*0.1*
*P. ovale*	5(3.8%)	2(8%)	3(2.8%)	*0.3*
*P. malariae*	2(1.5%)	1(4%)	1(0.9%)	*0.3*
*P. vivax*	0(0%)	0(0%)	0(0%)	*-*
***Coinfections***				
**Total**	19(14.4%)	10(40%)	11(10.3%)	***< 0.001***
Filaria	8(6.1%)	5(20%)	3(2.8%)	***0.004***
Geohelminths	10(7.6%)	7(28%)	3(2.8%)	***< 0.001***
Flukes	4(3%)	2(8%)	2(1.9%)	*0.1*
Protozoa	3(2.3%)	2(8%)	1(0.9%)	*0.1*
Virus	2(1.5%)	0(0%)	2(1.9%)	*-*
**Poliparasitized (+ 2)**	7(5.3%)	4(16%)	3(2.8%)	***0.02***
**Not coinfected**	**113(85.6%)**	12(48%)	96(92.9%)	***-***
***Symptoms***				
**Symptomatic**	71(91%)	8(66.7%)	63(95.5%)	***0.006***
Fever	49(62.8%)	7(25.3%)	42(63.6%)	*0.7*
Diarrhoea	12(15.4%)	1(8.3%)	11(16.7%)	*0.4*
Abdominal pain	17(21.8%)	4(33.3%)	12(18.2%)	*0.2*
Secondary symptoms ^1^	52(65.8%)	8(72.7%)	53(80.3%)	***0.5***
**Asymptomatic**	7(9%)	4(33.3%)	3(4.5%)	***0.006***
**Unknown**	**54(40.9%)**	**13(52%)**	**41(38.3%)**	***-***
***Diagnostic delay***				
< 30 days	93(76.2%)	6(33.3%)	87(83.6%)	***< 0.001***
30 days-3 months	18(14.8%)	7(38.8%)	11(10.6%)	***0.005***
> 3 months	11(9%)	5(27.7%)	6(5.8%)	***0.009***
**Unknown**	**10(7.6%)**	**7(28%)**	**3(2.8%)**	***-***
***Chemoprophylaxis***				
Correct	3(2.3%)	0(0%)	3(2.8%)	*-*
Incorrect	8(6.1%)	1(4%)	7(6.5%)	*0.6*
None	121(91.7%)	24(96%)	97(90.6%)	*0.3*
***Analytic alterations***				
Anaemia (Hb < 11.6 g/dL for women, Hb < 13.2 g/dLfor men)	47(35.6%)	11(44%)	36(33.6%)	0.3
Leucocytosis (> 4.8*10^3/µL)	72(54.5%)	13(52%)	61(57%)	0.6
Thrombocytopenia (< 130*10^3/µL)	0(0%)	0(0%)	0(0%)	-
GPT (> 45 U/L)	13(9.8%)	2(8%)	11(10.3%)	0.7
GOT (> 34 U/L)	20(15.2%)	3(12%)	17(15.9%)	0.6
Bilirubin (< 1.2 mg/dL)	18(13.6%)	2(8%)	17(15.9%)	0.3

*SMM: Submicroscopic Malaria*,* MM: Microscopical Malaria*,* VFR: Visiting Relatives and Friends*,* MRA: Migrants of recent arrival*,* T&B: Tourism and Business*,* Hb: Haemoglobin*,* GPT: Glutamine-pyruvate transaminase*,* GOT: Glutamine-oxaloacetate transaminase*

^*1*^*Secondary symptoms include vomiting*,* headache*,* cough*,* shivering*,* asthenia and general discomfort*

In addition to malaria, a concomitant imported disease was detected in 19 (14.4%) of the patients, 17 of them parasitic infections, mainly intestinal parasites, and two infections by imported viruses; one Dengue and other Chikungunya (Tables 1 and 2). All coinfection cases were concomitant with *falciparum*-malaria, except one that was a *malariae*-malaria with two coinfections *Schistosoma intercalatum* plus *Mansonella perstans*. MRA, with 10 patients (52.6%) was the most frequently coinfected group, showing higher rates of geohelminth and fluke infections. Eight (42.1%) patients were VFR, and the last one was a Spanish T&B who traveled to Equatorial Guinea for tourism and got malaria and *Loa loa* infection. By malaria type, SMM patients were the most frequently coinfected, especially with filaria and geohelminths.

Access to clinical manifestations was possible only in 78 out of 132 positive patients (59.1%). The most common symptom was fever in 49 patients (62.8%) followed by abdominal pain and diarrhoea (Tables 1 and 2).

Ninety-three patients (76.2%) were diagnosed within 30 days after they arrived in Spain. In nine patients the diagnosis delay was higher than 6 months, ranging from 6 months to 6 years and 4 months. Four of the patients were MM, two of them being caused by *P. ovale*, presenting symptoms, presumably due to relapses, and the other two by *P. falciparum*, being one of those asymptomatic, and the other with symptoms compatible with malaria but attributed to an abdominal abscess. The other five were SMM, all *P. falciparum* infections, two of them asymptomatic and in the other cases, clinical data were not available.

Only 11 patients (8.4%) reported having followed any chemoprophylaxis, but only three performed it correctly. In all three cases, the patients acquired *ovale*-malaria despite taking Proguanil-Atovaquone (2) and Doxycycline (1). The rest of the patients followed incorrect chemoprophylaxis, either due to incomplete dosing in seven cases or, in one case, chloroquine was employed as chemoprophylaxis in a Cameroon travel. Among these patients, seven were VFR and the last was a Spanish tourist.

**Fig. 2:**
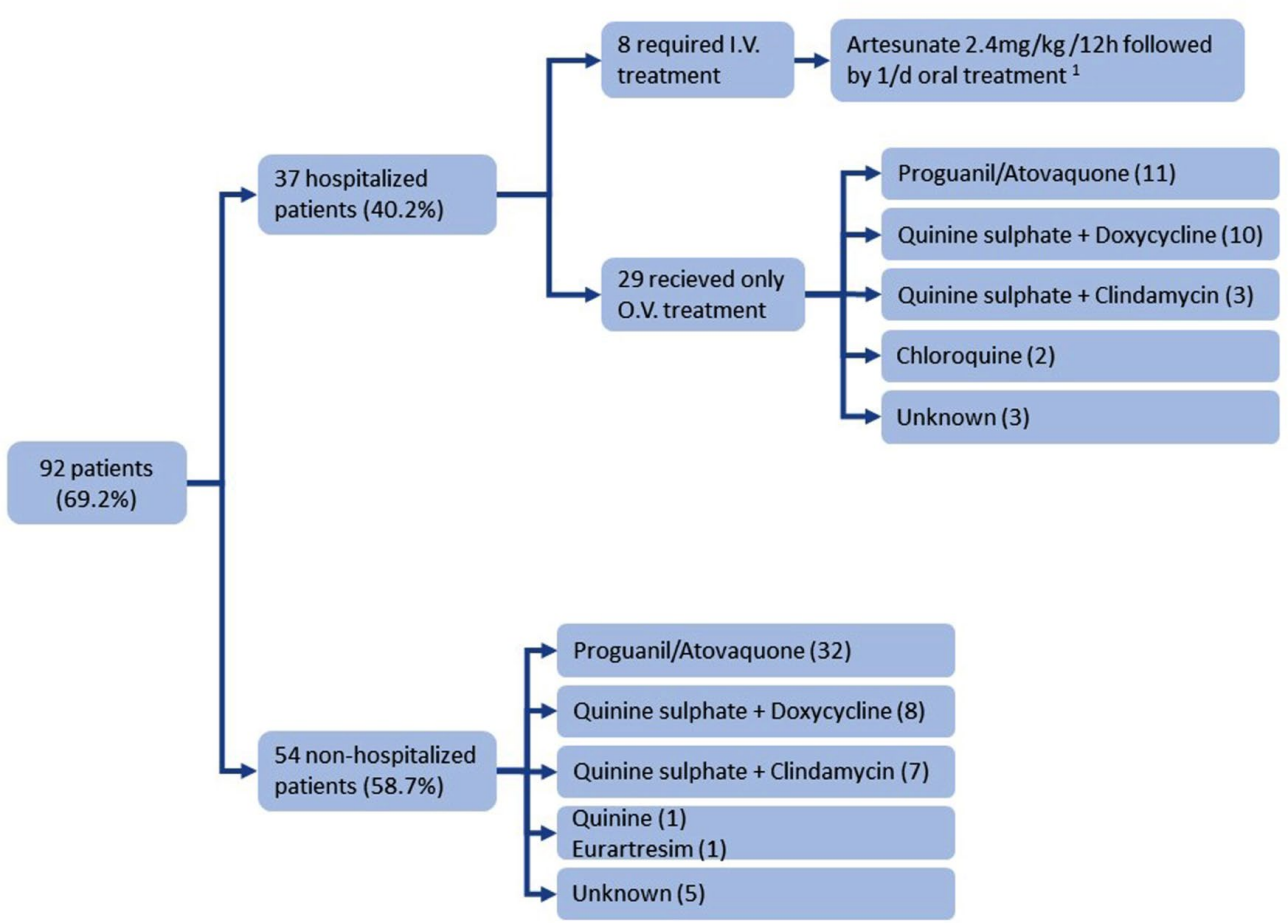
Distribution of the antimalarial treatments employed among our patients. I.V.: Intravenous via; O.V.: oral via

Accessing treatment data on patients´ history was possible only for 92 patients (69.7%), being this very variable but neither therapeutic failure nor drug resistance was observed (Fig. 2). Patients with malaria caused by *P. ovale* received conventional treatment followed by primaquine to eliminate the hypnozoites. Thirty-seven patients (40.2%) required hospitalisation, in eight cases (8.7%) with intravenous treatment. Only one patient required intensive care hospitalization, a Spanish T&B who visited Equatorial Guinea without taking prophylaxis, but no deaths from malaria were recorded, but none required intensive care hospitalization and no deaths from malaria were recorded.

## Discussion

This work provides a long-term review of imported malaria cases in Spain, one of the European countries where more cases are reported. Many aspects of the disease´s epidemiology, clinical presentation, diagnosis and management are analysed for the main types of patients faced in clinical practice according to their travel motivation. In addition, these key issues for malaria are compared between the most (MM) and least (SMM) frequent clinical presentations of malaria.

The main diagnostic method used in the hospital was microscopy, although it was the least sensitive (74.4%), as already demonstrated by other authors [16]. However, microscopy remains an important diagnostic tool, being able to estimate parasitemia and distinguish between the different *Plasmodium* species when an experienced observer performs it. ICT was the most rapid technique, showing a similar sensitivity to microscopy (78.2%), as was showed in previous studies [11, 17], but being handicapped by the inability to determine parasitemia and, in general, being only able to distinguish *P. falciparum* and not the other *Plasmodium* species for the lack of sensitivity. PCR resulted in the most sensitive technique (99.2%) allowing to distinguish between species. The disadvantage of molecular techniques is the fact that not being routinely carried out in many hospitals, and it is necessary to send them to reference laboratories, which delays results. In addition, despite being the most sensitive technique, one microscopy positive case resulted negative by PCR. False negatives PCR can occur, showing that not every method is perfect, and evidence for the need for combined diagnostic strategies to avoid false negatives as well as getting over the disadvantages of each technique by itself, allowing to make a reliable diagnosis in the shortest time [15].

Regarding intra-annual distribution of malaria cases, the end of summer in the northern hemisphere shows to be the year period in which more malaria suspicious patients attend to emergency department and more malaria diagnoses are established, this fact was also appreciated both in other Spanish and European studies [16, 18, 19], being mainly related to the return of patients travelling to their original countries in summer holidays.

In our study, VFR was the main group (69.7%) of malaria cases followed by MRA (25%) and finally T&B (5.3%), similar to other studies performed in Spain [20–22]. Instead, other studies carried out in Madrid (Spain) showed that MRA was the most important group, constituting close to 50% of malaria cases [23]. These differences may be due to the different populations assigned to each hospital by its influence area.

Most of cases got infected in sub-Saharan West Africa, being Equatorial Guinea the country where most patients got infected, which responded to the narrow relationship between Spain and its former colony, as shown in other national-wide studies [21, 22]. These data are clearly different from those obtained in other European countries, where a higher proportion of patients infected in central and East Africa was notified [24].

Mean age for the malaria patients in our study resulted to be 36 years old, with no significative difference from other studies [23, 24]. When analysing patient age according to their travel motive, MRA patients resulted to be the youngest group, with T&B being the oldest one, with these findings being consistent with other studies [14, 25].

The predominant aetiological agent of malaria was *P. falciparum* with a minority of *P. ovale* and *P. malariae* cases reported and with no *P. vivax* nor *P. knowlesi* cases. *P. falciparum* was also the main species in other European studies with patients being mainly infected in West Africa [19, 25, 26], with increased *P. vivax* cases being reported in other countries with a higher rate of central and east Africa infected patients [24].

Malaria endemic countries are also endemic for other tropical diseases, so co-infection is common. 14.4% of malaria patients present a concomitant tropical disease, with helminthiasis being the most frequent (7.6%), similar to what was found in a south Spain study carried out by Pousibet-Puerto et al. [14]. SMM (40%) resulted to be the most coinfected group, some studies have postulated that certain helminth infections, such as filariasis, can modulate the immune response to malaria favouring parasitemia control as is the case of SMM [27, 28].

MRA patients show fewer symptoms than VFR or T&B patients and symptoms such as fever were present in a lesser percentage than in the rest of the groups. The existence of malaria semi-immunity has been known for a long time, some studies have described how, after continued exposure to *Plasmodium*, this semi-immunity is acquired, protecting the host from developing severe and potentially fatal stage of the disease [14, 29–31]. This semi-immunity starts to wane with the absence of exposure, rendering that in VFR patients, living out of an endemic area for a long time, semi-immunity is gradually lost, leading to more symptomatic presentations [32]. Despite being most of the cases diagnosed in Spain MM, SMM is supposed an important percentage in this study, consisting in 18.9% of all cases, being lower than the 27.4% obtained in a study carried out in the South of Spain, in an area of high migratory pressure [14]. Instead, those percentages are quite higher than the 5% reported by other studies performed in Spain [13, 16].

As expected, and in general, most malaria cases, 76.2% in this study, are diagnosed in the first month after returning from endemic areas [14, 16, 33]. VFR resulted to be the earlier-diagnosed group probably because they recognize the symptoms, having previously suffered it or seeing cases around them in their countries of origin, and because of their loss of immunity, leaving them susceptible to more symptomatic malaria.

In nine patients the diagnosis was delayed more than 6 months because they did not present any symptoms during that period. These patients constitute malaria reservoirs in endemic areas and in non-endemic countries, where there are still anopheline vectors, they constitute an important risk of occurrence of autochthonous malaria cases and eventually a re-emergence of the illness [34, 35].

Only 2.3% (3) of the patients who took the chemoprophylaxis correctly got malaria caused by *P. ovale*. Drugs employed as chemoprophylaxis in these patients (Proguanil-Atovaquone and Doxycycline) are theoretically effective in avoiding acute malaria by eliminating blood stages of *Plasmodium* but only proguanil is effective against the infective sporozoite form. The most probable cause for the infection despite chemoprophylaxis is these drugs eliminate the blood forms and prevent primary infection, but the hypnozoites remain and relapse, in due time, when there is no longer drugs pressure. Several cases of *P. ovale* malaria related to chemoprophylaxis failure have been reported as being attributed to different causes just as drug resistance, incomplete chemoprophylaxis, or previous latent infection [36–38]. The rest of the patients who took chemoprophylaxis did it incorrectly or with ineffective drugs in the area due to resistance [39].

In our study, only one patient (0.75%) required ICU admission. This is a low ICU admission rate, but not significantly different from other South Madrid study [16]. At SOUH, ICU-admission criteria for malaria patients are slightly different from those postulated by WHO [10]. Attending to this last, all patients with a parasitemia higher than 10% must be ICU-admitted, but in our hospital, ICU admission does not depend on parasitemia but in the development of severe complications. Despite these stricter criteria, no malaria-related deceases have been notified in SOUH during the study period, demonstrating that all patients received effective medical attention. An additional reason explaining this fact are early diagnosis strategies carried out in our institution, making possible early treatment and avoiding complications.

In conclusion, it is observed that VFR individuals present malaria with greater symptoms that correspond to MM, unlike MRA individuals who present fewer or no symptoms and which in many cases is only detected by PCR (SMM), but these are those with a higher level of coinfection, possibly because it is the first time they come from an endemic area.

This study shows the need for establishing systems for VFRs to attend pre-travel consultations to increase the use of prophylaxis to reduce malaria imported risk, and in the case of MRA, screening for infectious diseases should be conducted upon their arrival to improve life quality of the patients and reduce the risk of autochthonous cases. Finally, we highlight two cases of malaria co-infection with Dengue and Chikungunya. These cases illustrate that the presence of malaria doesn’t rule out other imported diseases. Additionally, the presence of symptoms resembling malaria from another imported pathogen doesn’t exclude the possibility of malaria.

This study has some limitations, data concerning symptoms and malaria management were missing in some patients, as well as some analytical data. When analysing these parameters, patients with a lack of some of them were not taken into consideration.

## Data Availability

No datasets were generated or analysed during the current study.
